# Real-time ultrasound guidance for internal jugular vein catheterization in neonates: preliminary experience

**DOI:** 10.1186/cc9443

**Published:** 2011-03-11

**Authors:** M Di Nardo, F Stoppa, C Tomasello, C Cecchetti, M Marano, D Perrotta, E Pasotti, N Pirozzi

**Affiliations:** 1 Ospedale Pediatrico Bambino Gesù, Roma, Italy

## Introduction

Recent studies reported that real-time ultrasound guidance for internal jugular vein catheterization is useful in infants. However, this technique is sometimes difficult even for skilled physicians. The aim of our study is therefore to evaluate the success rate and the complication rate of this technique performed by ultrasound-trained pediatric intensivists in neonates.

## Methods

Fifteen consecutive term neonates (mean weight 3.9 ± 1.1 kg) needing a central venous access for intensive care treatment were prospectively studied for ultrasound-guided internal jugular vein cannulation. Patients' age, weight, time for cannulation, catheter size, central venous time permanence, success rate and complications rate were recorded.

## Results

Cannulation was successful in all 15 infants. The right internal jugular vein was used in 90% of the patients enrolled, while in the remaining 10% the left internal jugular vein was used. The overall complication rate was 22%. We had only one major complication (2%): lung pneumothorax. Minor complications were: multiple skin and vein punctures (9%), Seldinger wire kinking (7%) and venous hematomas (4%). Time required for complete cannulation was 8 ± 4.3 minutes, while the mean duration of the central venous catheter was 5 ± 5 days.

## Conclusions

Our results suggest that ultrasound assistance for central vein cannulation can be easily performed by well-trained physicians in neonates. Particular solutions (increase of the tilting angle of the bed, use of soft nitilon tip guide wire and the transfixation technique) can be sometimes requested to increase the success rate of our procedures. In accordance to these considerations, US-guided CVC placement should be probably considered as the first choice method for catheterization in infants.
